# Seasonal movements in caribou ecotypes of Western Canada

**DOI:** 10.1186/s40462-022-00312-x

**Published:** 2022-03-10

**Authors:** Jessica Theoret, Maria Cavedon, Troy Hegel, Dave Hervieux, Helen Schwantje, Robin Steenweg, Megan Watters, Marco Musiani

**Affiliations:** 1 Faculty of Environmental Design, University of Calgary, Calgary, AB T2N 1N4 Canada; 2 Yukon Department of Environment, Whitehorse, YT Y1A 2C6 Canada; 3 Fish and Wildlife Stewardship Branch, Alberta Environment and Parks, Grande Prairie, AB T8V 6J4 Canada; 4 Wildlife and Habitat Branch, Ministry of Forests, Lands, Natural Resource Operations and Rural Development, Government of British Columbia, 2080 Labieux Road, Nanaimo, BC V9T 6J9 Canada; 5 Pacific Region, Canadian Wildlife Service, Environment and Climate Change Canada, 5421 Robertson Road, Delta, BC V4K 3N2 Canada; 6 Land and Resource Specialist, 300 – 10003 110th Avenue, Fort St. John, BC V1J 6M7 Canada; 7 Department of Biological Sciences, Faculty of Science and Veterinary Medicine (Joint Appointment), University of Calgary, Calgary, AB T2N 1N4 Canada; 8 Present Address: Fish and Wildlife Stewardship Branch, Alberta Environment and Parks, 4999 98 Ave., Edmonton, AB T6B 2X3 Canada

**Keywords:** Migratory behaviour, Net square displacement, Seasonal ranges overlap, Plasticity, Caribou

## Abstract

**Background:**

Several migratory ungulates, including caribou, are dramatically declining. Caribou of the Barren-ground ecotype, which forms its own subspecies, are known to be mainly migratory. By contrast, within the Woodland subspecies, animals of the Boreal ecotype are known to be mainly sedentary, while those within the Northern and Central Mountain ecotypes to be partially migratory, with only some individuals migrating. Promotion of conservation actions (e.g., habitat protection) that are specific to both residents and migrants, as well as to the areas they frequent seasonally (which may be separate for migrants), requires distinguishing migration from other movement behaviours, which might be a challenge.

**Methods:**

We aimed at assessing seasonal movement behaviours, including migratory, resident, dispersing, and nomadic, for caribou belonging to the Barren-ground and Woodland subspecies and ecotypes. We examined seasonal displacement, both planar and altitudinal, and seasonal ranges overlap for 366 individuals that were GPS-collared in Northern and Western Canada. Lastly, we assessed the ability of caribou individuals to switch between migratory and non-migratory movement behaviours between years.

**Results:**

We detected migratory behaviour within each of the studied subspecies and ecotypes. However, seasonal ranges overlap (an index of sedentary behaviour) varied, with proportions of clear migrants (0 overlap) of 40.94% for Barren-ground caribou and 23.34% for Woodland caribou, and of 32.95%, 54.87%, and 8.86% for its Northern Mountain, Central Mountain, and Boreal ecotype, respectively. Plastic switches of individuals were also detected between migratory, resident, dispersing, and nomadic seasonal movements performed across years.

**Conclusions:**

Our unexpected findings of marked seasonal movement plasticity in caribou indicate that this phenomenon should be better studied to understand the resilience of this endangered species to habitat and climatic changes. Our results that a substantial proportion of individuals engaged in seasonal migration in all studied ecotypes indicate that caribou conservation plans should account for critical habitat in both summer and winter ranges. Accordingly, conservation strategies are being devised for the Woodland subspecies and its ecotypes, which were found to be at least partially migratory in this study. Our findings that migration is detectable with both planar and altitudinal analyses of seasonal displacement provide a tool to better define seasonal ranges, also in mountainous and hilly environments, and protect habitat there.

**Supplementary Information:**

The online version contains supplementary material available at 10.1186/s40462-022-00312-x.

## Background

Ungulate migration is an important natural phenomenon that affects both individual animals and ecological processes in the areas they seasonally frequent [1]. Yet, migratory behaviours are disappearing worldwide [2–4], so the study of ungulate migration is becoming increasingly important [5, 6]. The promotion of conservation actions that are specific to migrants, and to the areas they frequent [7], requires, as a first step, distinguishing migration from other movement behaviours, which is challenging [8, 9].

Migratory behaviour is commonly defined as the movement from one location to another and back, allowing animals to exploit seasonally- and geographically-variable resources (e.g., food, habitats in a broader sense, favorable climate, or breeding conditions) or avoid unfavorable conditions (e.g., predators, disease) [10]. In mountainous environments, these seasonal movements may also occur altitudinally (vertically) as opposed to planarly (horizontally; e.g., latitudinal migration in temperate and colder climates) [11]. Resident behaviour is instead characterized by comparatively short movements occurring within an area that is often frequented throughout an animal’s lifetime [12]. Other common movement behaviours include nomadism and dispersal, though the classification of these can also prove challenging [13]. Nomadic animals shift ranges continuously [14], whereas dispersing animals move from a natal range to a new range where they settle [15].

Seasonal movement behaviours are sometimes assessed for whole populations, which may result in a generalized, forced, and limiting description [16, 17]. Some species may be partially migratory, where within the same population only a fraction of individuals migrate [18, 19]. Appropriately defining and detecting seasonal movement behaviours of individuals, including in partially migratory populations [18, 20], may aid assessments of diversity within species and also affect conservation and management strategies in the areas frequented seasonally [21].

Individuals may also not be limited to one seasonal movement behaviour throughout their lifetime. Instead, they may switch behaviours between years, exhibiting behavioural plasticity [22]. For example, through the results of several studies on ungulates, it is now apparent that individuals can and do switch between migrant and resident movement behaviours [11, 23]. These switches in behaviour could be attributed to environmental changes from year to year, to learning, predation risk, competition, or a combination of all the above [10]. Individuals that are plastic in migratory behaviour are presumably more resilient to environmental or land-use changes [24]. Thus, determining (lack of) migratory plasticity may help identify at-risk populations (for example, those with a higher proportion of non-plastic individuals, whose seasonal ranges are experiencing human-caused habitat alterations) [25, 26].

Assessing seasonal movement behaviours is now aided by the use of animal-mounted sensors such as radio (e.g., Very High Frequency; VHF) and satellite (e.g., Geographic Positioning System; GPS) transmitters, which allow for data collection over considerable geographic scales [27, 28]. A common methodological approach is estimating seasonal ranges from the spatial clustering of telemetry locations during key times of the year, such as winter and summer [29]. Migrants are then classified based on the amount of seasonal range overlap [30, 31]. Though this approach to distinguishing migratory behaviour is straightforward, it may not detect altitudinal migration [32]. A newer approach is conducting Net Squared Displacement (NSD) analyses to examine seasonal planar displacement of individuals [33]. Additionally, recent adaptations of the NSD approach by Spitz et al. (2017) [13] have provided a method for analyzing altitudinal movement behaviours, as well.

**Fig. 1:**
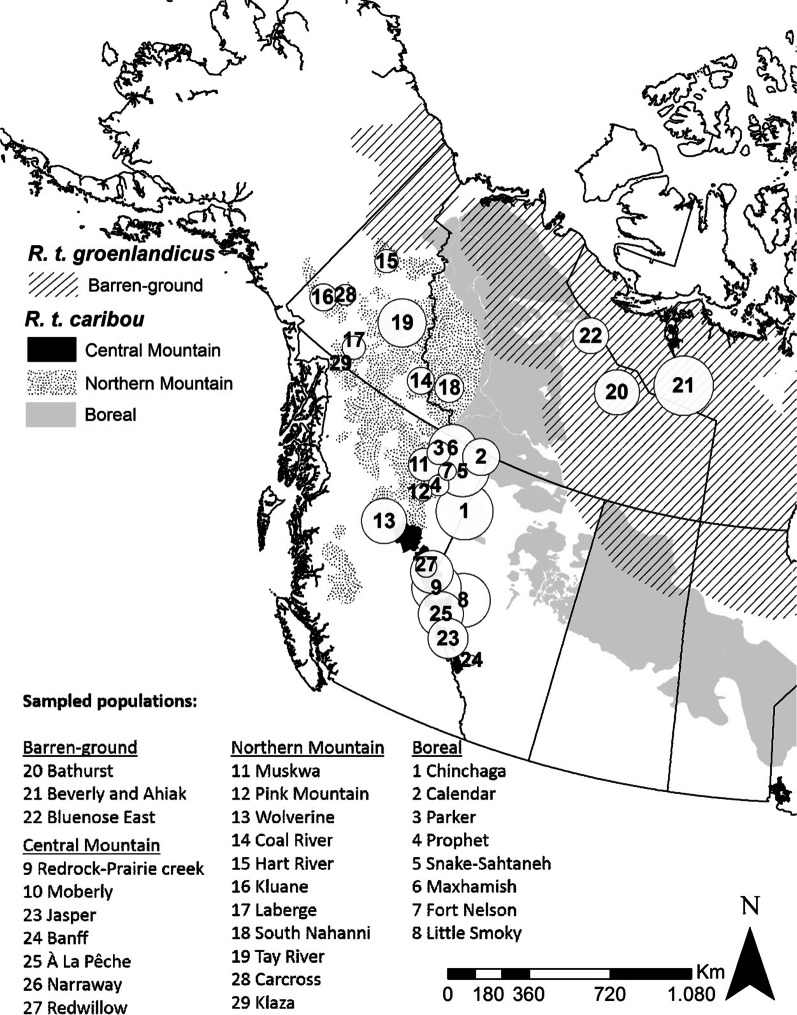
Caribou sampled in Northwestern Canada for seasonal movement analyses. Black numbered circles indicate sampled populations (also referred to as “herds”, as they might not be genetically or ecologically distinct). Circles are proportional to sample size per population (mean = 13.07, SD = 9.57, range 1–33). Grey-scale polygons show the distribution of subspecies and ecotypes: diagonal black lines represent the Barren-ground subspecies (*R. t. groenlandicus*); black-dots, black, and light gray represent the Northern Mountain, Central Mountain, and Boreal ecotypes, respectively, within the Woodland caribou subspecies (*R. t. caribou*). A summary table of our sample of monitored caribou individuals is in Additional file 4: Data file S1

Caribou (*Rangifer tarandus*) are known for having one of the longest-range migrations among ungulates [34], and are drastically declining across their distributional range, which also includes North America [35–37]. The presence and type of migratory behaviour are known to vary across caribou subspecies, ecotypes, and populations (i.e., caribou is partially migratory) [38, 39]. Caribou of the Barren-ground ecotype, which forms its own subspecies (*R. t. groenlandicus—*Fig. 1), are known to be mainly migratory, performing long-distance migrations (~ 300 km one way) [34, 40]. By contrast, within the Woodland subspecies (*R. t. caribou*), animals of the Boreal ecotype are known to be mainly sedentary, while those within the Northern and Central Mountain ecotypes (like all Mountain ecotypes of Western Canada) to be partially migratory, with some individuals only migrating and performing short distance migration (up to 70 km one way [38, 40]). Yet, formal and quantitative assessments of partial migration are lacking for caribou subspecies and ecotypes living in Western Canada. Furthermore, recent studies indicate the presence of plasticity in seasonal movement behaviours of Mountain and Alaskan caribou, with animals switching from migrant to resident behaviour or changing the location of calving grounds across years [41, 42]). Therefore, studying and quantifying migration and seasonal movement plasticity in endangered caribou is becoming a priority.

We aimed at assessing the seasonal movement behaviours of caribou belonging to the Barren-ground and Woodland subspecies and ecotypes. We examined seasonal displacement, both planar and altitudinal, and seasonal range overlap for individuals that were GPS-collared in Northern and Western Canada (Fig. 1). Lastly, we assessed the ability of caribou individuals to switch between migratory and non-migratory movement behaviours in different years.

## Methods

### Study area

Our study included caribou of the Barren-ground and Woodland subspecies belonging to 29 populations across Alberta, British Columbia, Northwest Territories, Nunavut, and Yukon, Canada (Fig. 1). The sampled Barren-ground ecotype, which forms its own subspecies, resided within the southern arctic tundra, taiga plain, and shield ecozones of the Northwest Territories and Nunavut. These ecozones are characterized by seasonally variable snow and ice cover with predominantly treeless and flat areas, or rolling hills where the land is dominated by both wetlands and shrublands [43]. The sampled caribou of the Woodland subspecies belonged to three ecotypes: Northern Mountain, Central Mountain, and Boreal. Northern Mountain caribou predominantly resided in the boreal, montane, and taiga cordillera ecozones of the Rocky Mountains [43]. Central Mountain caribou occupied a mix of flat and mountainous areas, characteristic of boreal plain and montane cordillera ecozones (respectively) [44]. Boreal caribou inhabited boreal and taiga plain ecozones, which have little variability in elevation [45, 46].

### Data collection and screening

Female caribou were radio-collared by government staff or contractors of Alberta, British Columbia, Northwest Territories, Nunavut, and Yukon between 1998 and 2018, each following their respective government’s standardized permitting, animal care, and handling procedures. The collaring of females was decided by the governing bodies, since females are considered as a first monitoring priority for conservation. Females are also ideal to define seasonal movements in caribou [47], as they show fidelity to areas used during a fixed calving period [39]. Collars varied with respect to their duration (minimum = 2 months, maximum = 6 years) and were equipped with a fix interval ranging from hourly to daily. Following Cagnacci et al. (2015) [17], we filtered and standardized telemetry data for each animal to obtain daily locations. After screening procedures, the data set contained 230,791 locations for 366 unique individuals: 64 individuals belonged to the Barren-ground subspecies and 302 individuals belonged to three ecotypes within the Woodland subspecies (Northern Mountain, n_individuals_ = 92; Central Mountain, n_individuals_ = 79; Boreal, n_individuals_ = 131).

### Planar and altitudinal seasonal displacement analyses

**Fig. 2:**
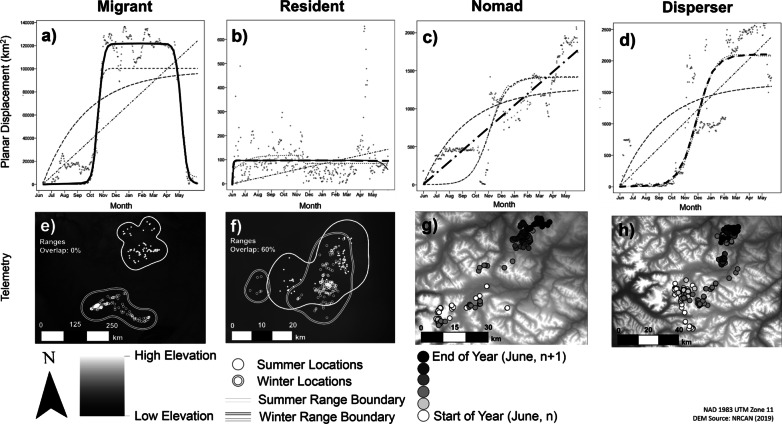
Seasonal movement patterns monitored with GPS-collars for caribou in Northwestern Canada. Panels **a**–**d** show net square displacements for caribou individuals classified as Migrant, Resident, Nomad, or Disperser (a description of patterns for Mixed-Migrants is in Methods). The displacement is best represented by a bell curve (marked with a continuous line above) when the animal is migratory (**a**). An animal is classified as Resident (**b)** when the best fitting line quickly reaches an asymptote. Whereas Nomad (**c**) and Disperser (**d)** are characterized by diagonal or “s” shaped lines, respectively. Solid lines represent the best fitted models, while other lines represent unsupported models of seasonal movement. Panels **e**–**h** show winter and summer locations for the same animals above. **e** shows a complete separation of seasonal ranges, typical of migratory animals, while the animal in **f** has ranges overlap and is considered Resident. For Nomads (**g**) and Dispersers (**h**), locations are provided in shades of grey, representing time of year (ranges not provided as not established). A detailed description of NSD models representing seasonal movement behaviours is in Additional file 1: Method S1

We examined planar displacement of caribou by conducting Net Squared Displacement (NSD) analyses within the *R* package *MigrateR*, which allows the classification of individuals as either Migrant, Mixed Migrant (i.e., individuals returning to a different location than the initial one), Resident, Disperser, or Nomad (see methodology in Additional file 1: Method S1) [13, 33]. With these analyses, the distance (in square kilometers, km^2^; i.e., the squared displacement) from a starting telemetry location to all subsequent locations for an individual during a year is graphed (Fig. 2a–d). A family of a priori regression models, each representing the curve indicating a different movement behaviour (see Additional file 1: Method S1, for a description of models) are then fit to the distribution. Then, the best model (if fitting a Migrant, Mixed Migrant, Resident, Disperser, or Nomad curve) is chosen through Akaike’s Information Criteria (AIC) [48].

We conducted analyses for both a winter and a calving start date (see Additional file 1: Method S2), as some caribou may show fidelity to calving grounds used in late spring (typically Barren-ground caribou), while others to wintering areas (some Mountain ecotypes; [49]). We also utilized the option Relative Net Squared Displacement (rNSD), which is ideal for start date selection [13]. We set the parameter called ρ to 30 (indicating that a caribou had to spend at least 30 days in a range to be considered migratory) following recommendations established for ungulates [17, 50]. We conducted NSD-planar analyses for different subsets of behavioural categories: (a) inclusion of all movement categories (Migrant, Mixed Migrant, Resident, Disperser, and Nomad; (b) removal of the nomadic category (following the methods of Peters et al., 2017 [50]); or (c) category limitation to Migrant or Resident behaviour.

This study’s Woodland caribou were located in mountain ranges and some of them, particularly within Northern and Central Mountain ecotypes, are known to migrate altitudinally [49]. We therefore used *MigrateR* to also examine altitudinal displacement and classify individuals as Migrant, Resident, or Disperser (classification as Nomad and Mixed Migrant is not offered by this type of analysis) [13]. Similarly to NSD-planar analyses, NSD-altitudinal analyses were conducted for two start dates. Finally, we conducted NSD-altitudinal analyses for all movement categories and also removed the Disperser category for categorization to exclusively Migrant or Resident behaviour.

### Seasonal ranges overlap and comparison of methods

We calculated an index of overlap (IO) between winter and summer ranges frequented by individual caribou, with IO ranging from 0 to 1 (higher and lower values indicating resident and migratory behaviour, respectively; Fig. 2e–h). We defined summer (1 July—15 September) and winter (1 December—30 April) seasons following McDevitt et al. (2009) [47] and only used individuals with at least 30 locations per season [51]. For each animal, we estimated seasonal utilization distributions (UD) using the *kernelUD* function (with reference bandwidth) within the *adehabitatHR* package [52] in R version 3.5. We then derived range contour polygons from the 95% fixed-kernel isopleth. Finally, we determined the IO for each animal following McDevitt et al. (2009) [47]:

$${\text{IO}} = \left[ {2A_{12} /\left( {A_{1} + A_{2} } \right)} \right]$$

where *A*_12_ is the area of overlap (km^2^) between the summer and winter 95% isopleths, and *A*_1_ and *A*_2_ are the areas (km^2^) of the summer and winter 95% isopleths for the animal, respectively. Individuals with 0% overlap were considered “clear migrants”, as this is an intuitive threshold to distinguish migratory movements from other seasonal movements.

To compare results obtained with the two methods (NSD vs. ranges overlap), we ran the Kruskal–Wallis one-way analysis of variance, within IBM SPSS Statistics [53], to test whether seasonal ranges overlap (IO) was different between seasonal movement categories obtained with NSD planar or with altitudinal displacement analyses (above). When significance was detected (*p* value < 0.05), we then used the Mann–Whitney U test to determine pairwise differences between seasonal movement categories.

### Assessing behavioural plasticity

We assessed individual switches in seasonal movements between years (i.e., a form of behavioural plasticity) based on the NSD categorization of movement obtained from either planar or altitudinal displacement analyses. We obtained a plasticity metric (P) for each caribou using the following equation:

$${\text{P}} = \Delta C_{Years} /\left( {C_{Years} - 1} \right)$$

where *C*_*Years*_ is the total number of years of data, and ∆*C*_*Years*_ is the number of categorization switches between sequential years. We calculated P among Resident, Nomad, Disperser, and Migrant plus Mixed-Migrant behaviours exhibited in a given year (note that firm categorizations as Mixed Migrant are disputed in the literature and could represent Migrants) [33]. We also calculated P for Resident vs. Migrant binary categorizations. A plasticity metric equal to 1 indicates switches in behaviour categorization between all sequential years (indicating that the animal was completely plastic), while a plasticity metric of 0 indicates no switch.

## Results

### Migratory behaviour detected within each subspecies and ecotype

Results of planar displacement analyses conducted with a calving start date indicated that a large proportion of animals within each subspecies and ecotype was classified as Migrant (Table 1). In particular, when classification was restricted to only two types of seasonal movement behaviour (Migrant or Resident), 90% of Barren-ground caribou and 59% of Woodland were classified as Migrant. Within the Woodland subspecies, proportions of migratory animals varied by ecotype: 70%, 59%, and 55% of Migrants were detected for Northern Mountain, Central Mountain, and Boreal caribou, respectively. When we considered all behavioural types, a large proportion of migrants were actually classified as Mixed Migrant, where individuals made a return movement to a different location than their initial one. Sedentary animals were sparse, varying from 2% of Barren-ground to 4% of Boreal caribou. High proportions of caribou were Dispersers, especially within ecotypes of the Woodland subspecies (Table 1). The removal of the Nomadic category from analyses provided similar classifications of animals (Additional file 2: Table S1).

**Table 1:** Metrics of seasonal ranges overlap and planar and altitudinal displacement for caribou subspecies (Barren-ground and Woodland), and, separately, for the studied Woodland ecotypes (in italics)

Subspecies/*ecotype*	Migration distance for migrants (km)		Ranges overlap			Planar displacement							Altitudinal displacement				
	Median	Mean	% clear mig.^a^	Median	Mean	Binary^b^		All categories^b^					Binary^b^		All categories^b,c^		
						% mig	% res	% mig	% mixmig	% res	% disp.	% nomad	% mig	% res	% mig	% disp.	% res
Barren-ground	*279.75*	318.47	40.94	3	8	90, 62	10, 38	43, 8	53, 63	2, 2	2, 22	0, 5	NA	NA	NA	NA	NA
Woodland	49.71	47.71	23.34	14	20	59, 61	41, 39	26, 11	46, 60	3,6	19, 18	6, 5	70, 74	30, 26	60, 63	33, 32	7, 5
Northern Mountain	*40.17*	*56.02*	32.95	0	11	*70, 70*	*30, 30*	*37, 12*	*44, 68*	*0, 6*	*18, 11*	*1, 3*	*79, 84*	*21, 16*	*58, 79*	*41, 20*	*1, 1*
Central Mountain	*54.94*	*54.96*	54.87	0	14	*59, 74*	*41, 26*	*33, 18*	*42, 64*	*2,2*	*20, 16*	*5, 0*	*68, 79*	*32, 21*	*64, 74*	*32, 23*	*4, 3*
Boreal	*23.61*	*28.75*	8.86	22	25	*55, 50*	*45, 50*	*20, 9*	*49, 54*	*4, 6*	*18, 23*	*9, 8*	*67, 66*	*33, 34*	*60, 51*	*29, 41*	*11, 8*

^a^No overlap detected (0%)

^b^First and second numbers indicate results of displacement analyses conducted with calving or winter start dates, respectively

^c^For altitudinal displacement analyses only three movement categories can be assessed

When planar displacement analyses were conducted with a winter start date, behavioural classification changed compared to analyses conducted with a calving start date. In particular, more Residents (38%) and Dispersers (22%) were detected for Barren-ground caribou when using either the binary or the all-categories classification, respectively.

**Fig. 3:**
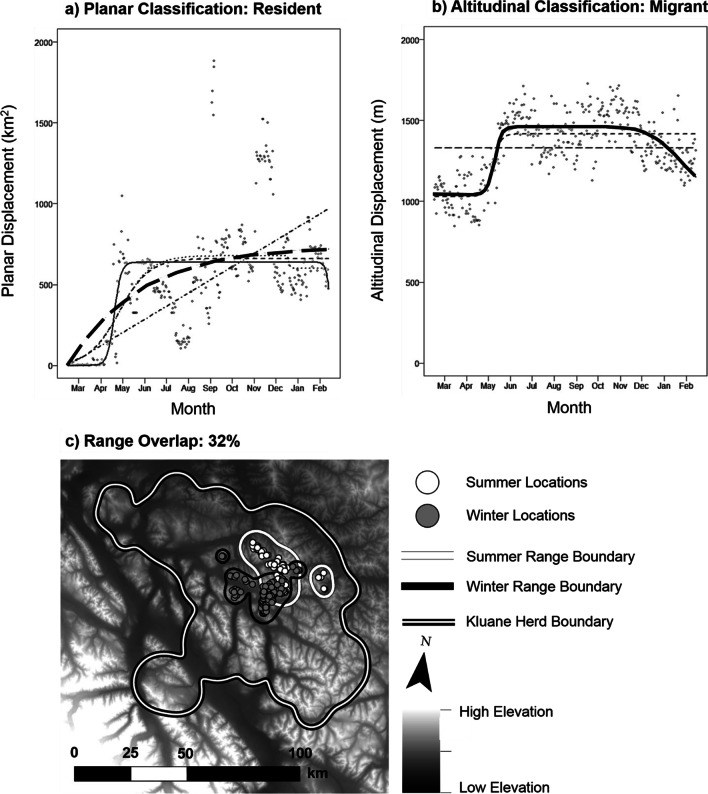
Planar vs. altitudinal displacement in caribou. Planar (**a**) and altitudinal (**b)** displacement plots implying classification of the same animal as Resident or Migrant, respectively. Panel **c** shows seasonal ranges for the same caribou

Results of altitudinal displacement analyses, which were conducted for Woodland caribou only, indicated a higher proportion of Migrants compared to planar displacement analyses, particularly when using a binary classification (Fig. 3, Table 1 and Additional file 2: Table S1). Within Northern Mountain, Central Mountain, and Boreal ecotypes, 84%, 79%, and 66% of animals were Migratory (respectively; percentages reported for winter start date). Differences in seasonal behaviour type classification were negligible when using a winter or calving start date.

Mean values of migration distance (one way), calculated for migratory animals only, were 318.47 km for Barren-ground caribou (range = 140.71–986.39) and 49.71 km for Woodland caribou (range = 6.31–129.93). Within Woodland caribou, the mean value for the Northern Mountain ecotype was 56.02 km (range = 8.48–129.94) and that for the Central Mountain ecotype was 54.97 km (range = 15.90–81.68). Finally, the mean migration distance for the Boreal ecotype was 28.75 km (range = 6.30–76.36).

### Seasonal ranges overlap varying between subspecies and ecotypes

The median value of seasonal ranges overlap for Barren-ground caribou was 3%, with a proportion of clear migrants (IO = 0) of 40.94% (Table 1). The median ranges overlap for Woodland caribou was 14%, with a proportion of clear migrants (IO = 0) of 23.34%. Within the Woodland subspecies, seasonal ranges overlapped by 0% (median) for both the Northern and Central Mountain ecotypes, and by 22% (median) for the Boreal ecotype. The proportions of clear migrants were 32.95%, 54.87%, and 8.86% for the Northern Mountain, Central Mountain, and Boreal ecotypes, respectively.

### Differences in overlap between seasonal movement categories

**Fig. 4:**
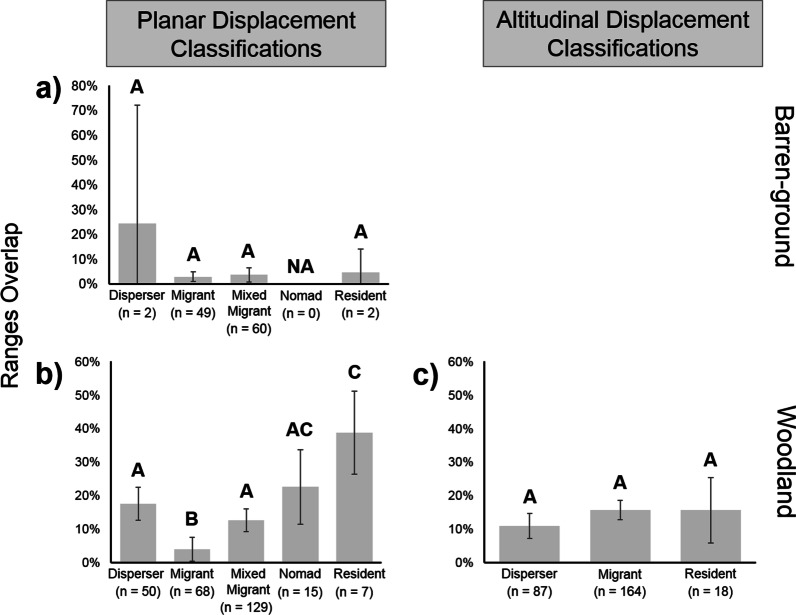
Comparisons of seasonal ranges overlap between seasonal movement categories obtained with displacement analyses. Panel **a** shows seasonal ranges overlap (%) for Barren-ground caribou individuals classified with planar displacement analyses as Dispersers, Migrants, Mixed Migrants, or Residents (low sample sizes for Dispersers and Residents may invalidate statistical comparisons). Panel **b** shows the same information for Woodland caribou individuals (Nomad were also detected). Panel **c** also shows this information for Woodland caribou individuals that were instead classified with altitudinal displacement analyses (this analysis was not conducted for Barren-ground caribou). The upper-case letters A/B/C and their combinations denote significant differences. Sample sizes are reported (n), as well as 95% confidence intervals

We examined differences in seasonal ranges overlap between movement categories obtained with planar (Additional file 2: Table S2) or altitudinal displacement analyses (Additional file 2: Table S3). Within the Barren-ground subspecies, we did not detect differences in seasonal ranges overlap between Dispersers, Migrants, Mixed Migrants, and Residents (Fig. 4a), indicating that this subspecies was more homogeneous than the Woodland subspecies, which included multiple ecotypes in this study. Differences in overlap were detected within the Woodland subspecies (planar displacement analyses; Fig. 4b). Migrants had lower overlap (median = 3.94, c.i. = 3.54) than Residents (median = 38.79, c.i. = 12.40), Nomadic individuals (median = 22.58, c.i. = 11.12), Mixed Migrants (median = 12.65, c.i. = 3.40), and Dispersers (median = 17.47, c.i. = 4.94), with these last two further differentiated from Residents. Within the Woodland ecotypes, differences between seasonal movement categories were detected only for Northern Mountain caribou (Additional file 3: Fig. S1). When comparing overlap of seasonal movement categories obtained with altitudinal displacement analyses (Additional file 3: Fig. S1), differences were detected only for Northern Mountain caribou, in which Migrants had lower overlap (median = 7.78, c.i. = 5.71) than Dispersers (median = 0, c.i. = 5.09).

### Seasonal movement plasticity detected in subspecies and ecotypes

**Fig. 5:**
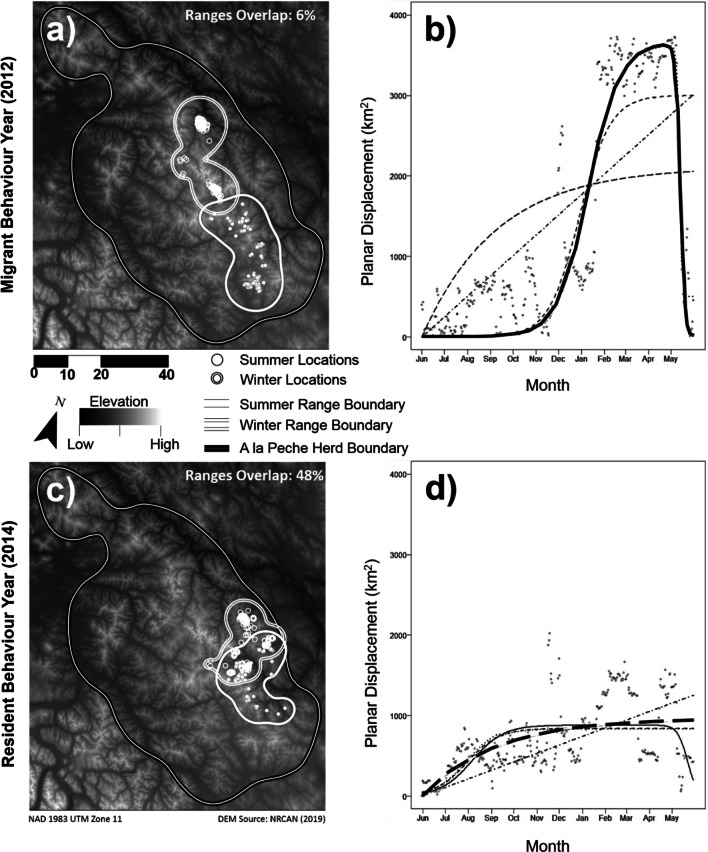
Behavioural plasticity in caribou. Panels **a** and **b** show seasonal ranges and a displacement plot (respectively) of an individual performing migration during 2012. Panels **c** and **d** show seasonal ranges and displacement plot (respectively) for the same animal being Resident in 2014

Seasonal movement plasticity, calculated as a metric varying from 0 (fixed behaviour) to 1 (entirely plastic behaviour), was detected within each studied subspecies and ecotype, particularly when examining switches between Migratory and Resident behaviours only, and vice-versa (Fig. 5, Additional file 2: Table S4). Plasticity, between Migratory and Resident behaviours only, determined with planar displacement analyses was 0.19 and 0.47 for the Barren-ground and Woodland subspecies, respectively. We detected approximately an equal amount of switches from Migrant to Resident and from Resident to Migrant. Within the Woodland subspecies, plasticity values for Northern Mountain, Central Mountain, and Boreal caribou were 0.33, 0.4, and 0.52, respectively. Plasticity of seasonal movements determined with altitudinal displacement analyses was 0.4 for the Woodland subspecies, and 0.31, 0.6, and 0.57 for its Northern Mountain, Central Mountain, and Boreal ecotypes, respectively.

## Discussion

### Detection of migration in both Barren-ground and Woodland caribou

In this study, we detected seasonal migration in Barren-ground caribou and in surprisingly large numbers of individuals belonging to the Woodland subspecies, where residential behaviour was expected to be the norm [39, 54, 55]. Our results supported conventional knowledge that Barren-ground caribou are mostly migratory, exhibiting seasonal returns to their calving grounds [56–58]. By contrast, when using a winter start date in our analyses, we detected fewer migratory animals, which could be expected since the winter ground locations for Barren-ground caribou have been known to fluctuate relative to calving ground locations [59, 60].

However, contrary to current knowledge [39, 54, 55], we also detected a high number of migratory animals in the Woodland subspecies, which was confirmed with both altitudinal and planar analyses of seasonal displacement, although such migrations were of shorter range. This last finding suggests that Woodland caribou practice altitudinal migration as much as planar migration, quantitatively supporting what has been previously observed qualitatively for this subspecies [61–63]). The ranges of the planar migrations that we detected were several folds greater for Barren-ground than for Woodland caribou and in line with the literature [34, 40]. These scale considerations also apply to seasonal ranges overlap analyses (discussed below), where a similar value of overlap has different implications for the vast ranges of barren ground caribou. Therefore, our results for Woodland caribou might not have conservation implications as substantial as for Barren-ground caribou, where conservation planners are investing significant resources to delineate and protect the extensive migratory routes [64]. Our study focused on distances of migration. However, animals, caribou included, also have minor shifts between seasonally used core areas. Future studies could evaluate and compare migratory to other seasonal range shifts and relate those to environmental drivers.

For Woodland caribou, analyses conducted with either a winter or calving start date indicated equivalent proportions of seasonal movement behaviours (and similar proportions of migrants), supporting the observation that wintering and calving grounds are equally established [49], and contrary to the more variable wintering grounds of Barren-ground caribou.

Seasonal displacement analyses conducted for the Woodland ecotypes showed that the proportion of migratory animals was higher for Northern and Central Mountain caribou than for Boreal caribou. Mountain ecotype migrants were numerous when examining altitudinal displacement in particular, aligning well with current understandings that these caribou can migrate from forested wintering areas to alpine or subalpine areas, where they calve in the spring and remain for the summer [65–68]. Boreal migrants were relatively less numerous, but still more than expected based on the literature, which describes them as largely sedentary [38, 69, 70]. Boreal caribou may inhabit relatively flat stands of boreal forest across Canada [71]. However, this study’s Boreal caribou were from the foothills or hilly regions of Western Canada, so the availability of resources may be more spatially (altitudinally and geographically) and temporally segregated, offering migrants the opportunity to access these seasonally [72].

Our assessment of seasonal ranges overlap further supported results of the displacement analyses. Overlap values were lower for Barren-ground animals, supporting the notion that this subspecies is largely migratory [73]. By contrast, higher values were detected for Woodland animals—and for the Boreal ecotype in particular—where residential behaviour is known to occur more frequently [39]. On the other hand, seasonal overlap was lower for Northern and Central Mountain caribou, suggesting that migratory behaviour was prevalent within these two ecotypes, again an original finding. Firm delineation of caribou into geographically separate ecotypes is currently being debated in the literature [38], and our results could in theory have been influenced by misclassifications of some caribou base upon provenance. This study was the first to analyze caribou GPS data with new biostatistical approaches to determine migration, and this could explain partially why migrants were not detected in several previous studies. Overall, seasonal overlap values were lower than expected within each studied ecotype, contradicting the generalization that Woodland caribou are primarily non-migratory [39, 54, 55].

### Difficulties in categorizing seasonal movement behaviours in caribou

Like other authors, we found that seasonal movement categorization through displacement analyses can be time-consuming and open to interpretation [17]. For example, some of our dispersers may have instead been residents or migrants, even after taking precautions to reduce such an issue (i.e., we defined a minimum time of range occupancy) [33, 50]. To avoid misclassification, researchers have suggested visually inspecting displacement outputs for final categorization [13, 74, 75], which may not be feasible when analyzing a large number of individuals, as we did. Overall, using multiple methods and comparing results is advisable [17, 76, 77]; as such, we conducted and evaluated several variations of planar and altitudinal displacement analyses, and also examined ranges overlap. Furthermore, we tested seasonal overlap differences between movement categories obtained with displacement analyses, which, to our knowledge, has not been accomplished before.

Various studies that used seasonal ranges overlap analyses to detect migrants and residents followed the traditional definition of ungulate migration, which requires allopatric seasonal ranges and considers any amount of overlap greater than 0% indicative of resident behaviour [30, 31, 78]. However, like others [17], we found that individuals categorized as Migrant or Mixed Migrant through displacement analyses had varying degrees of seasonal range overlap. We suggest that seasonal displacement and ranges overlap analyses may be considered complementary methods for describing movement behaviour. Finally, it is also possible that in some circumstances intermediate seasonal movement behaviours occur [74, 79, 80], which will defy any classification effort. In view of our results, streamlined displacement models examining three major classes of seasonal movement behaviours (Migrant, Resident, and others) could perhaps provide useful results. Finally, the development of integrative approaches, such as combining altitudinal and planar displacement analyses, might also be useful in discerning between seasonal movements.

### Tendency to switch seasonal movement behaviours and conservation of caribou

Though recent studies have detected plasticity in the migratory behaviour of large herbivores [81–83], there remains a lack of understanding surrounding behavioural plasticity in caribou, which weakens conservation efforts [38, 41]. Future studies could look at the natural, climatic and anthropic drivers of switches in seasonal movement behaviours, and perhaps try to correct them if related to human impacts. Our results indicated that plasticity of migratory movements across years was present in both subspecies of caribou, though at a larger scale for Woodland individuals and in particular for its Central Mountain ecotype. The levels of seasonal movement plasticity we found were comparable to those described for elk, an ungulate that is known for flexibility in movement patterns [84, 85]. Individuals that switch between migratory and resident behaviours are presumably at a greater advantage, as they may be more resilient to environmental or land-use changes [50, 86]. Nonetheless, as recently found [41], caribou that switch behaviour from migrant to resident forcibly by human-caused habitat alterations of seasonal ranges and migratory routes may have lower survival. Our findings did not support a pattern in switches from migratory to resident behaviour or *vice-versa*, and detection of temporal trends in plasticity would require a longitudinal study. Further studies could test whether plasticity in seasonal movements is influenced by natural factors (related to caribou, other species or the environment) and by human factors as they vary by area.

Furthermore, migratory behaviour could also have a genetic component, as shown by Cavedon et al. (2019) [87] and potential genetic determination may diminish the resilience and survival of caribou. In support to this argument, Cavedon et al. (2022) [88] documented genes determining migratory behaviour in caribou, which could further impact the species, possibly by permanent loss of the genetic drivers, even just by drift, in some populations already at low numbers. We failed to detect any temporal trends in plasticity. Regardless, our unexpected findings of marked seasonal movement plasticity in caribou indicate that this phenomenon should be better studied to understand the resilience of this endangered species to habitat and climatic changes.

## Conclusions

Our findings that a substantial proportion of individuals engage in seasonal migration in all studied ecotypes indicate that caribou conservation plans should account for critical habitat (sensu [71]) in both summer and winter ranges. The practice is fairly established for the widely known migratory Barren-ground subspecies [89], but conservation strategies are still being devised for the Woodland subspecies, also including its Northern Mountain, Central Mountain, and Boreal ecotypes [90, 91], which were found to be at least partially migratory in this study. Our findings that migration is detectable with both planar and altitudinal analyses of seasonal displacement provide a tool to better define seasonal ranges, also in mountainous and hilly environments as well as to protect habitat there. Delineation of seasonal ranges is a necessary next step to perform habitat selection and/or connectivity analyses, to enable conservation planners to preserve caribou habitat throughout the year.

## Supplementary Information

**Additional file 1**. Supplemental methods.

**Additional file 2**. Supplemental figures.

**Additional file 3**. Supplemental tables.

**Additional file 4**. Supplemental data.

## Data Availability

Spatial environmental data are freely available at https://open.canada.ca. Raw movement data for caribou is not publicly available as Canadian provincial and federal governments monitor this Species at Risk. Other datasets used and/or analyzed during the current study are available from the corresponding author upon request.

## Abbreviations

NSD: Net square displacement
IO: Index of overlap

## References

[1] Bauer S, Hoye BJ (2014). Migratory animals couple biodiversity and ecosystem functioning worldwide. Science.

[2] Harris G, Thirgood S, Hopcraft JG, Cromsigt JP, Berger J (2009). Global decline in aggregated migrations of large terrestrial mammals. Endanger Species Res.

[3] Tucker MA, Böhning-Gaese K, Fagan WF, Fryxell JM, Van Moorter B, Alberts SC, Ali AH, Allen AM, Attias N, Avgar T, Bartlam-Brooks H (2018). Moving in the anthropocene: global reductions in terrestrial mammalian movements. Science.

[4] Kauffman MJ, Cagnacci F, Chamaillé-Jammes S, Hebblewhite M, Hopcraft JG, Merkle JA, Mueller T, Mysterud A, Peters W, Roettger C, Steingisser A (2021). Mapping out a future for ungulate migrations. Science.

[5] Runge CA, Martin TG, Possingham HP, Willis SG, Fuller RA (2014). Conserving mobile species. Front Ecol Environ.

[6] Allen AM, Singh NJ (2016). Linking movement ecology with wildlife management and conservation. Front Ecol Evol.

[7] Kubelka V, Sandercock BK, Székely T, Freckleton RP (2021). Animal migration to northern latitudes: environmental changes and increasing threats. Trends Ecol Evol.

[8] Mueller T, Fagan WF (2008). Search and navigation in dynamic environments—from individual behaviors to population distributions. Oikos.

[9] Mueller T, Olson KA, Dressler G, Leimgruber P, Fuller TK, Nicolson C, Novaro AJ, Bolgeri MJ, Wattles D, DeStefano S, Calabrese JM (2011). How landscape dynamics link individual-to population-level movement patterns: a multispecies comparison of ungulate relocation data. Glob Ecol Biogeogr.

[10] Dingle H, Drake VA (2007). What is migration?. Bioscience.

[11] Berg JE, Hebblewhite M, St Clair CC, Merrill EH (2019). Prevalence and mechanisms of partial migration in ungulates. Front Ecol Evol.

[12] Roshier DA, Reid JR (2003). On animal distributions in dynamic landscapes. Ecography.

[13] Spitz DB, Hebblewhite M, Stephenson TR (2017). ‘MigrateR’: extending model-driven methods for classifying and quantifying animal movement behavior. Ecography.

[14] Teitelbaum CS, Mueller T (2019). Beyond migration: causes and consequences of nomadic animal movements. Trends Ecol Evol.

[15] Johnson ML, Gaines MS (1990). Evolution of dispersal: theoretical models and empirical tests using birds and mammals. Annu Rev Ecol Syst.

[16] Holyoak M, Casagrandi R, Nathan R, Revilla E, Spiegel O (2008). Trends and missing parts in the study of movement ecology. PNAS.

[17] Cagnacci F, Focardi S, Ghisla A, Van Moorter B, Merrill EH, Gurarie E, Heurich M, Mysterud A, Linnell J, Panzacchi M, May R (2015). How many routes lead to migration? Comparison of methods to assess and characterize migratory movements. J Anim Ecol.

[18] Chapman BB, Brönmark C, Nilsson JÅ, Hansson LA (2011). The ecology and evolution of partial migration. Oikos.

[19] Buchan C, Gilroy JJ, Catry I, Franco AM (2020). Fitness consequences of different migratory strategies in partially migratory populations: a multi-taxa meta-analysis. J Anim Ecol.

[20] Lundberg P (1988). The evolution of partial migration in birds. Trends Ecol Evol.

[21] Hertel AG, Niemelä PT, Dingemanse NJ, Mueller T (2020). A guide for studying among-individual behavioral variation from movement data in the wild. Mov Ecol.

[22] Stamps JA (2016). Individual differences in behavioural plasticities. Biol Rev.

[23] Eggeman SL, Hebblewhite M, Bohm H, Whittington J, Merrill EH (2016). Behavioural flexibility in migratory behaviour in a long-lived large herbivore. J Anim Ecol.

[24] Xu W, Barker K, Shawler A, Van Scoyoc A, Smith JA, Mueller T, Sawyer H, Andreozzi C, Bidder OR, Karandikar H, Mumme S (2021). The plasticity of ungulate migration in a changing world. Ecology.

[25] Faille G, Dussault C, Ouellet JP, Fortin D, Courtois R, St-Laurent MH, Dussault C (2010). Range fidelity: the missing link between caribou decline and habitat alteration?. Biol Conserv.

[26] Lafontaine A, Drapeau P, Fortin D, St-Laurent MH (2017). Many places called home: the adaptive value of seasonal adjustments in range fidelity. J Anim Ecol.

[27] Nathan R, Getz WM, Revilla E, Holyoak M, Kadmon R, Saltz D, Smouse PE (2008). A movement ecology paradigm for unifying organismal movement research. PNAS.

[28] Hughey LF, Hein AM, Strandburg-Peshkin A, Jensen FH (2018). Challenges and solutions for studying collective animal behaviour in the wild. Philos Trans R Soc.

[29] Lichti NI, Swihart RK (2011). Estimating utilization distributions with kernel versus local convex hull methods. J Wildl Manag.

[30] Mysterud A (1999). Seasonal migration pattern and home range of roe deer (Capreolus capreolus) in an altitudinal gradient in southern Norway. J Zool.

[31] Cagnacci F, Focardi S, Heurich M, Stache A, Hewison AM, Morellet N, Kjellander P, Linnell JD, Mysterud A, Neteler M, Delucchi L (2011). Partial migration in roe deer: migratory and resident tactics are end points of a behavioural gradient determined by ecological factors. Oikos.

[32] Bastille-Rousseau G, Potts JR, Yackulic CB, Frair JL, Ellington EH, Blake S (2016). Flexible characterization of animal movement pattern using net squared displacement and a latent state model. Mov Ecol.

[33] Bunnefeld N, Börger L, van Moorter B, Rolandsen CM, Dettki H, Solberg EJ, Ericsson G (2011). A model-driven approach to quantify migration patterns: individual, regional and yearly differences. J Anim Ecol.

[34] Joly K, Gurarie E, Sorum MS, Kaczensky P, Cameron MD, Jakes AF, Borg BL, Nandintsetseg D, Hopcraft JG, Buuveibaatar B, Jones PF (2019). Longest terrestrial migrations and movements around the world. Science.

[35] Vors LS, Boyce MS (2009). Global declines of caribou and reindeer. Glob Change Biol.

[36] Festa-Bianchet M, Ray JC, Boutin S, Côté SD, Gunn A (2011). Conservation of caribou (Rangifer tarandus) in Canada: an uncertain future. Can J Zool.

[37] Plante S, Dussault C, Richard JH, Côté SD (2018). Human disturbance effects and cumulative habitat loss in endangered migratory caribou. Biol Conserv.

[38] Committee on the Status of Endangered Wildlife in Canada (COSEWIC). Designatable Units for Caribou (Rangifer tarandus) in Canada. Committee on the Status of Endangered Wildlife in Canada. Ottawa; 2011.

[39] Bergerud AT, Luttich SN, Camp L. Return of caribou to Ungava (Vol. 50). McGill-Queen's Press-MQUP; 2007

[40] Berger J (2004). The last mile: how to sustain long-distance migration in mammals. Conserv Biol.

[41] Williams SH, Steenweg R, Hegel T, Russell M, Hervieux D, Hebblewhite M (2021). Habitat loss on seasonal migratory range imperils an endangered ungulate. Ecol Solut Evid.

[42] Fullman TJ, Person BT, Prichard AK, Parrett LS (2021). Variation in winter site fidelity within and among individuals influences movement behavior in a partially migratory ungulate. PLoS ONE.

[43] Commission for Environmental Cooperation (CEC): ecological regions of North America: toward a common perspective. Québec; 1997.

[44] Ireland G, Petropoulos GP (2015). Exploring the relationships between post-fire vegetation regeneration dynamics, topography and burn severity: a case study from the Montane Cordillera Ecozones of Western Canada. Appl Geogr.

[45] Schultz J (2005). The ecozones of the world—the ecological divisions of the geosphere.

[46] Ecosystem Classification Group (ECG). Ecological regions of the northwest territories – Taiga Plains. Department of Environment and Natural Resources, Government of the Northwest Territories, Yellowknife, NT, Canada; 2007

[47] McDevitt AD, Mariani S, Hebblewhite M, Decesare NJ, Morgantini L, Seip D, Weckworth BV, Musiani M (2009). Survival in the Rockies of an endangered hybrid swarm from diverged caribou (Rangifer tarandus) lineages. Mol Ecol.

[48] Burnham KP, Anderson DR (2002). Model selection and multimodel inference—a practical information-theoretic approach.

[49] Committee on the Status of Endangered Wildlife in Canada (COSEWIC). Assessment and status report on the Caribou Rangifer tarandus, Northern Mountain population, Central Mountain population and Southern Mountain population in Canada. Committee on the Status of Endangered Wildlife in Canada. Ottawa; 2014

[50] Peters W, Hebblewhite M, Mysterud A, Spitz D, Focardi S, Urbano F, Morellet N, Heurich M, Kjellander P, Linnell JD, Cagnacci F (2017). Migration in geographic and ecological space by a large herbivore. Ecol Monogr.

[51] Girard I, Ouellet JP, Courtois R, Dussault C, Breton L (2002). Effects of sampling effort based on GPS telemetry on home-range size estimations. J Wildl Manag.

[52] Calenge C (2006). The package “adehabitat” for the R software: a tool for the analysis of space and habitat use by animals. Ecol Modell.

[53] IBM Corporation: IBM SPSS Statistics for Windows, Version 27.0. Armonk, NY: IBM Corporation; 2020.

[54] Bergerud AT (1988). Caribou, wolves and man. Trends Ecol Evol.

[55] Bergerud AT (1996). Evolving perspectives on caribou population dynamics, have we got it right yet?. Rangifer.

[56] Gunn A, Miller FL (1986). Traditional behaviour and fidelity to caribou calving grounds by barren-ground caribou. Rangifer.

[57] Gurarie E, Hebblewhite M, Joly K, Kelly AP, Adamczewski J, Davidson SC, Davison T, Gunn A, Suitor MJ, Fagan WF, Boelman N (2019). Tactical departures and strategic arrivals: divergent effects of climate and weather on caribou spring migrations. Ecosphere.

[58] Joly K, Gurarie E, Hansen DA, Cameron MD (2021). Seasonal patterns of spatial fidelity and temporal consistency in the distribution and movements of a migratory ungulate. Ecol Evol.

[59] Ferguson MA, Messier F (2000). Mass emigration of arctic tundra caribou from a traditional winter range: population dynamics and physical condition. J Wildl Manag.

[60] Ferguson MA, Gauthier L, Messier F (2001). Range shift and winter foraging ecology of a population of Arctic tundra caribou. Can J Zool.

[61] Edmonds EJ (1988). Population status, distribution, and movements of woodland caribou in west central Alberta. Can J Zool.

[62] Seip DR, McLellan B, Hummel M, Ray J (2008). Mountain caribou. Caribou and the north: a shared future.

[63] Couturier S, Otto RD, Côté SD, Luther G, Mahoney SP (2010). Body size variations in caribou ecotypes and relationships with demography. J Wildl Manag.

[64] Conference of Management Authorities. Recovery Strategy for Barren-ground Caribou (Rangifer tarandus groenlandicus) in the Northwest Territories. Conference of Management Authorities, Yellowknife, NT; 2020.

[65] Boonstra R, Sinclair AR (1984). Distribution and habitat use of caribou, rangifer tarandus caribou, and moose, alces alces andersoni, in the Spatsizi Plateau Wilderness area. British Columbia Can Field Nat.

[66] Saher DJ, Schmiegelow FK (2005). Movement pathways and habitat selection by woodland caribou during spring migration. Rangifer.

[67] Gustine DD, Parker KL, Lay RJ, Gillingham MP, Heard DC (2006). Interpreting resource selection at different scales for woodland caribou in winter. J Wildl Manag.

[68] Nobert BR, Milligan S, Stenhouse GB, Finnegan L (2016). Seeking sanctuary: the neonatal calving period among central mountain woodland caribou (Rangifer tarandus caribou). Can J Zool.

[69] Brown GS, Mallory FF, Rettie J (2003). Range size and seasonal movement for female woodland caribou in the boreal forest of northeastern Ontario. Rangifer.

[70] Wilson KS, Pond BA, Brown GS, Schaefer JA (2019). The biogeography of home range size of woodland caribou Rangifer tarandus caribou. Divers Distrib.

[71] Environment Canada. Scientific assessment to inform the identification of critical habitat for Woodland Caribou (Rangifer tarandus caribou), Boreal Population, in Canada: 2011 update. Ottawa, Ontario, Canada; 2011.

[72] Watters M, DeMars C (2016). There and back again: one Caribou’s (Rangifer tarandus) migratory behaviour hints at genetic exchange between designatable units. Can Field-Nat.

[73] Leclerc M, Leblond M, Le Corre M, Dussault C, Côté SD (2021). Determinants of migration trajectory and movement rate in a long-distance terrestrial mammal. J Mammal.

[74] Mysterud A, Loe LE, Zimmermann B, Bischof R, Veiberg V, Meisingset E (2011). Partial migration in expanding red deer populations at northern latitudes—a role for density dependence?. Oikos.

[75] Bischof R, Loe LE, Meisingset EL, Zimmermann B, Van Moorter B, Mysterud A (2012). A migratory northern ungulate in the pursuit of spring: jumping or surfing the green wave?. Am Nat.

[76] Rolandsen CM, Solberg EJ, Sæther BE, Moorter BV, Herfindal I, Bjørneraas K (2017). On fitness and partial migration in a large herbivore-migratory moose have higher reproductive performance than residents. Oikos.

[77] Martin J, Tolon V, Morellet N, Santin-Janin H, Licoppe A, Fischer C, Bombois J, Patthey P, Pesenti E, Chenesseau D, Saïd S (2018). Common drivers of seasonal movements on the migration-residency behavior continuum in a large herbivore. Science.

[78] Craighead JJ, Atwell G, O'Gara BW (1972). Elk migrations in and near Yellowstone National Park. Wildl Monogr.

[79] Edelhoff H, Signer J, Balkenhol N (2016). Path segmentation for beginners: an overview of current methods for detecting changes in animal movement patterns. Mov Ecol.

[80] Gurarie E, Bracis C, Delgado M, Meckley TD, Kojola I, Wagner CM (2016). What is the animal doing? Tools for exploring behavioural structure in animal movements. J Anim Ecol.

[81] Gurarie E, Cagnacci F, Peters W, Fleming CH, Calabrese JM, Mueller T, Fagan WF (2017). A framework for modelling range shifts and migrations: asking when, whither, whether and will it return. J Anim Ecol.

[82] Couriot O, Hewison AM, Saïd S, Cagnacci F, Chamaillé-Jammes S, Linnell JD, Mysterud A, Peters W, Urbano F, Heurich M, Kjellander P (2018). Truly sedentary? The multi-range tactic as a response to resource heterogeneity and unpredictability in a large herbivore. Oecologia.

[83] Peters W, Hebblewhite M, Mysterud A, Eacker D, Hewison AM, Linnell JD, Focardi S, Urbano F, De Groeve J, Gehr B, Heurich M (2019). Large herbivore migration plasticity along environmental gradients in Europe: life-history traits modulate forage effects. Oikos.

[84] Geist V, Thomas JW, Toweill DE (1982). Adaptive behavioral strategies. Elk of North America: ecology and management.

[85] Boyce MS (1991). Migratory behavior and management of elk (Cervus elaphus). Appl Anim Behav Sci.

[86] Sawyer H, Merkle JA, Middleton AD, Dwinnell SP, Monteith KL (2019). Migratory plasticity is not ubiquitous among large herbivores. J Anim Ecol.

[87] Cavedon M, Gubili C, Heppenheimer E, vonHoldt B, Mariani S, Hebblewhite M, Hegel T, Hervieux D, Serrouya R, Steenweg R, Weckworth BV (2019). Genomics, environment and balancing selection in behaviourally bimodal populations: the caribou case. Mol Ecol.

[88] Cavedon M, vonHoldt B, Hebblewhite M, Hegel T, Heppenheimer E, Hervieux D, Mariani S, Schwantje H, Steenweg R, Theoret J, Watters M, Musiani M (2022). Genomic legacy of migration in endangered caribou. PLoS Genet.

[89] Conference of Management Authorities. Recovery Strategy for Barren-ground Caribou (Rangifer tarandus groenlandicus) in the Northwest Territories. Yellowknife: Conference of Management Authorities; 2020.

[90] Canada E (2014). Recovery Strategy for the Woodland Caribou, Southern Mountain population (Rangifer tarandus caribou) in Canada. Species at risk act recovery strategy series.

[91] Environment and Climate Change Canada. Report on the Progress of Recovery Strategy Implementation for the Woodland Caribou (Rangifer tarandus caribou), Boreal population in Canada for the Period 2012–2017. Species at risk act recovery strategy series. Ottawa: Environment and Climate Change Canada; 2017.

