# Supplementary material for: Seasonal movements in caribou ecotypes of Western Canada
Source: Mov Ecol. 2022 Mar 10;10:12. doi: 10.1186/s40462-022-00312-x (PMC8908644; doi:10.1186/s40462-022-00312-x)
Supplement: Supplementary file 1 — Additional file 1. Supplemental methods. [file 40462_2022_312_MOESM1_ESM.docx]

**Additional file 1. Supplemental methods**

**Method S1. Categories of seasonal movements assessed with Net Square Displacement (NSD) analyses**

Our study relied on definitions of Migrant, Mixed Migrant, Resident, Nomad, and Disperser behavioural “categories” that, in the context of NSD analysis, were first described by Bunnefeld *et al.* (2011). Each category represents a pre-defined movement pattern relative to a stable starting location. A migrant behaviour would exhibit minimal displacement at first, followed by an increase as the animal moves into a new range, and finally a decrease as the animal returns to its initial location. The NSD model representing this pattern is a double sigmoid or s-shaped function (i.e., an extension of the logistic curve), where asymptotic heights, which represent distances of migration, have both equal value (i.e., representing the return to the exact same location). Mixed Migrant behaviour is similar to Migrant behaviour in that displacement decreases as the individual makes a return movement to its initial starting location, but it is not complete and slightly off from the starting point. The NSD model representing this pattern is also a double sigmoid, but the two asymptotes are not identical. Resident behaviour, by contrast, is signified by a relatively low displacement, with the animal remaining close to the start location throughout the duration of the year. In NSD analyses, therefore, Resident behaviour is represented by an asymptotic regression model. Nomadic behaviour is indicated by a displacement that steadily increases from the start date with no return, while a Dispersing behaviour exhibits a displacement that increases but then stabilizes as the animal establishes a range in a new location. Nomadic and Dispersing behaviour are therefore represented by a linear and a single sigmoid model, respectively.

**Method S2. Definition of winter and calving dates for NSD analyses**

According to Bunnefeld *et al*. (2011), the selected start date should be a time that an individual, if migratory, is within a stable seasonal range (e.g., summer or winter) and thus exhibits relatively minimal movement in terms of distance. Barren-ground and Woodland caribou are within their respective winter ranges from November to April (Simpson, Terry, & Hamilton, 1997; Ferguson & Elkie, 2004; Nicholson, Arthur, Horne, Garton, & Del Vecchio, 2016). To ensure that caribou were within their stable winter range for the selected first date of capture, we selected February 15 as the start date, or the first date of capture if it fell between February 15 and April 15 (i.e., all days between February 15 and April 15 were considered within the winter period for caribou; Cagnacci *et al*., 2015). To ensure the yearly telemetry period was long enough to capture the signal of a return migratory movement to the winter range after approximately one year’s time, the end of the year was considered to be February 14 of the following year, or the end of monitoring, if monitoring occurred after December 1 (i.e., all dates between December 1 and February 14 were also within the winter period for caribou; Cagnacci *et al*., 2015).

The calving season is notably shorter than the winter season; thus, the start date for the period of calving needed to be precise. Additionally, the dates of synchronistic calving differ between Barren-ground and Woodland caribou, with Woodland caribou calving earlier in the summer (i.e., mid-May to mid-June) (COSEWIC, 2011; COSEWIC, 2014; Norbert *et al*. 2016; Pond *et al*. 2016) than Barren-ground caribou (i.e., between June 1 and June 20) (Gunn *et al*. 1986; Chen *et al*., 2014; Nicholson *et al*. 2016). Following the rationale for selecting the winter start date, we ensured the first date of capture for the calving season was close to the median calving date (when caribou would be within their calving range) by allowing for a 5-day window prior to and following the median calving date for each subspecies. Therefore, the start date was set to May 27, or the first date of capture if it fell between May 27 and June 6 for Woodland caribou (i.e., all dates between May 27 and June 6 were considered within the calving period for Woodland caribou). For Barren-ground caribou, the start date was set to June 5, or the first date of capture if it fell between June 5 and June 15 (i.e., all dates between June 5 and June 15 were considered within the calving period for Barren-ground caribou). The end of the telemetry year was considered the day before the start date in the following year, or up to 2.5 months prior to the end of the year (March 12 and March 21 for Woodland and Barren-ground caribou, respectively).

References for Supplemental methods

Bunnefeld N, Börger L, van Moorter B, Rolandsen CM, Dettki H, Solberg EJ, Ericsson G. A model‐driven approach to quantify migration patterns: individual, regional and yearly differences. J Anim Ecol. 2011;80:466-476.

Simpson K, Terry E, Hamilton D. Toward a mountain caribou management strategy for British Columbia–habitat requirements and subpopulation status. British Columbia Ministry of Environment, Lands and Parks Wildlife Working Report WR-90, Victoria. British Columbia, Canada. 1997.

Ferguson SH, Elkie PC. Seasonal movement patterns of woodland caribou (Rangifer tarandus caribou). Journal of Zoology. 2004;262:125-34.

Nicholson KL, Arthur SM, Horne JS, Garton EO, Del Vecchio PA. Modeling caribou movements: seasonal ranges and migration routes of the Central Arctic Herd. PLoS One. 2016;11:e0150333.

Cagnacci F, Focardi S, Ghisla A, Van Moorter B, Merrill EH, Gurarie E, Heurich M, Mysterud A, Linnell J, Panzacchi M, May R. How many routes lead to migration? Comparison of methods to assess and characterize migratory movements J Anim Ecol. 2015;54-68.

Committee on the Status of Endangered Wildlife in Canada (COSEWIC). Designatable Units for Caribou (Rangifer tarandus) in Canada. Committee on the Status of Endangered Wildlife in Canada. Ottawa; 2011.

Committee on the Status of Endangered Wildlife in Canada (COSEWIC). Assessment and status report on the Caribou Rangifer tarandus, Northern Mountain population, Central Mountain population and Southern Mountain population in Canada. Committee on the Status of Endangered Wildlife in Canada. Ottawa; 2014.

Nobert BR, Milligan S, Stenhouse GB, Finnegan L. Seeking sanctuary: the neonatal calving period among central mountain woodland caribou (Rangifer tarandus caribou). Can J Zool. 2016;94:837-851.

Pond BA, Brown GS, Wilson KS, Schaefer JA. Drawing lines: Spatial behaviours reveal two ecotypes of woodland caribou. Biological Conservation. 2016;194:139-48.

Gunn A, Miller FL. Traditional behaviour and fidelity to caribou calving grounds by barren-ground caribou. Rangifer. 1986;1:151-158.

Chen W, White L, Adamczewski JZ, Croft B, Garner K, Pellissey JS, Clark K, Olthof I, Latifovic R, Finstad GL. Assessing the impacts of summer range on Bathurst caribou’s productivity and abundance since 1985. Natural Resources. 2014;28.

Nicholson KL, Arthur SM, Horne JS, Garton EO, Del Vecchio PA. Modeling caribou movements: seasonal ranges and migration routes of the Central Arctic Herd. PLoS One. 2016;11(4):e0150333.
