# Supplementary material for: Seasonal movements in caribou ecotypes of Western Canada
Source: Mov Ecol. 2022 Mar 10;10:12. doi: 10.1186/s40462-022-00312-x (PMC8908644; doi:10.1186/s40462-022-00312-x)
Supplement: Supplementary file 2 — Additional file 2. Supplemental figures. [file 40462_2022_312_MOESM2_ESM.docx]

**Additional file 2. Supplemental tables**

**Table S1** Percentages of caribou classified with planar-displacement analyses as Migrant, Mixed Migrant, Resident, or Disperser (after removal of the Nomadic category from analyses).

| Subspecies/*ecotype* | % Mig. ^a^ | % Mixmig. | % Res. | % Disp. |
| --- | --- | --- | --- | --- |
| Barren-ground | 43, 8 | 53, 63 | 2, 2 | 2, 27 |
| Woodland | 26, 11 | 46, 60 | 6, 8 | 21, 21 |
| *Northern Mountain* | *20, 9* | *49, 54* | *10, 11* | *21, 26* |
| *Central Mountain* | *33,18* | *42, 64* | *0, 1* | *25, 17* |
| *Boreal* | *37, 12* | *44, 68* | *0, 12* | *18, 18* |
| ^a^ First and second numbers indicate results of displacement analyses conducted with a calving or winter start date, respectively. | | | | |

**Table S2** Comparisons of ranges overlap between seasonal movement categories obtained with planar displacement analyses, with *p* values of Mann-Whitney U test pairwise (significant values in bold).

|  | Seasonal movement category (median; c.i.) | Disperser | Migrant | Mixed Migrant | Nomad | Resident |
| --- | --- | --- | --- | --- | --- | --- |
| Barren-ground | Disperser (17.47; 4.94) | *p value* of Kruskal Wallis test=0.896;  therefore pairwise tests not conducted | | | | |
|  | Migrant (3.94; 3.54) |  |  |  |  |  |
|  | Mixed Migrant (12.65; 3.40) |  |  |  |  |  |
|  | Nomad (22.58; 11.12) |  |  |  |  |  |
|  | Resident (38.79; 12.40) |  |  |  |  |  |
| Woodland | Disperser (17.47; 4.94) | - | **0.002** | 0.481 | 0.511 | **0.038** |
|  | Migrant (3.94; 3.54) | - | - | **0.002** | **0.025** | **0.001** |
|  | Mixed Migrant (12.65; 3.40) | - | - | - | 0.361 | **0.026** |
|  | Nomad (22.58; 11.12) | - | - | - | - | 0.340 |
|  | Resident (38.79; 12.40) | - | - | - | - | - |
| *Northern Mountain* | Disperser (22.03; 10.09) | - | **0.002** | 0.061 | NA | 0.189 |
|  | Migrant (0; 3.63) | - | - | **0.048** | NA | **0.027** |
|  | Mixed Migrant (0; 6.03) | - | - | - | NA | 0.096 |
|  | Nomad (NA; NA) | - | - | - |  | NA |
|  | Resident (48.38; NA) | - | - | - | - | - |
| *Central Mountain* | Disperser (0; 11.46) | *p value* of Kruskal Wallis test=0.103;  therefore pairwise tests not conducted | | | | |
|  | Migrant (0; 6.95) |  |  |  |  |  |
|  | Mixed Migrant (8.59; 6.91) |  |  |  |  |  |
|  | Nomad (0; NA) |  |  |  |  |  |
|  | Resident (NA; NA) |  |  |  |  |  |
| *Boreal* | Disperser (17.47; 6.62) | *p value* of Kruskal Wallis test=0.289;  therefore pairwise tests not conducted | | | | |
|  | Migrant (18.71; 5.17) |  |  |  |  |  |
|  | Mixed Migrant (22.60; 4.50) |  |  |  |  |  |
|  | Nomad (34.37; 11.25) |  |  |  |  |  |
|  | Resident (30.06; 17.78) |  |  |  |  |  |
|  |  | | | | | |
|  |  |  |  |  |  |  |

**Table S3** Comparison of ranges overlap between all movement categories obtained with altitudinal displacement analyses.

|  | Behavioural type  (median; c.i.) | Disperser ^a^ | Migrant | Resident | Disperser |
| --- | --- | --- | --- | --- | --- |
| Woodland | Disperser (10.95; 3.79) | *p value* of Kruskal Wallis test=0.107;  Mann-Whitney U test not conducted | | | |
|  | Migrant (15.64; 2.89) |  |  |  |  |
|  | Resident (15.62; 9.70) |  |  |  |  |
| *Northern Mountain* | Disperser (0; 5.09) | - | **0.023** | NA | NA |
|  | Migrant (7.78; 5.71) | - | - | NA | NA |
|  | Resident(NA: NA) | - | - |  | NA |
| *Central Mountain* | Disperser (0; 7.80) | *p value* of Kruskal Wallis test=0.518;  Mann-Whitney U test not conducted | | | |
|  | Migrant (0; 5.68) |  |  |  |  |
|  | Resident (0; NA) |  |  |  |  |
| *Boreal* | Disperser (19.16; 4.43) | *p value* of Kruskal Wallis test=0.851;  Mann-Whitney U test not conducted | | | |
|  | Migrant (21.58; 3.80) |  |  |  |  |
|  | Resident (19.33; 10) |  |  |  |  |
|  | ^a^ *p values* obtained with Mann-Whitney U test are indicated between pairwise movement categories; significant values are in bold | | | | |

**Table S4** Behavioural plasticity in caribou subspecies and ectypes**.**

| Subspecies/  *ecotypes* | plasticity between  planar-displacement categories ^a^ | | plasticity between  altitudinal-displacement categories ^a^ | |
| --- | --- | --- | --- | --- |
|  | binary ^b^ | all categories ^c^ | binary ^b^ | all categories ^c^ |
| Barren ground | 0.19/0.09 | 0.04 | NA | NA |
| Woodland | 0.47/0.21 | 0.44 | 0.4/0.2 | 0.52 |
| *Northern Mountain* | 0.33/0.18 | 0.25 | 0.18/0.7 | 0.31 |
| *Central Mountain* | 0.4/0.20 | 0 | 0.67/0.67 | 0.6 |
| *Boreal* | 0.52/0.22 | 0.54 | 0.45/0.2 | 0.57 |
| ^a^ plasticity is a metric varying from 0 (fixed behaviour) to 1 (entirely plastic behaviour)  ^b^ only switches between Migrant and Resident were examined. Second value indicates plasticity (switches from migrant to resident behaviour)  ^c^ switches between all movement types (Migrant, Resident, Disperser, and Nomad) were examined | | | | |
