# Supplementary material for: Seasonal movements in caribou ecotypes of Western Canada
Source: Mov Ecol. 2022 Mar 10;10:12. doi: 10.1186/s40462-022-00312-x (PMC8908644; doi:10.1186/s40462-022-00312-x)
Supplement: Supplementary file 3 — Additional file 3. Supplemental tables. [file 40462_2022_312_MOESM3_ESM.docx]

**Additional file 3. Supplemental figures**

**Fig. S1**


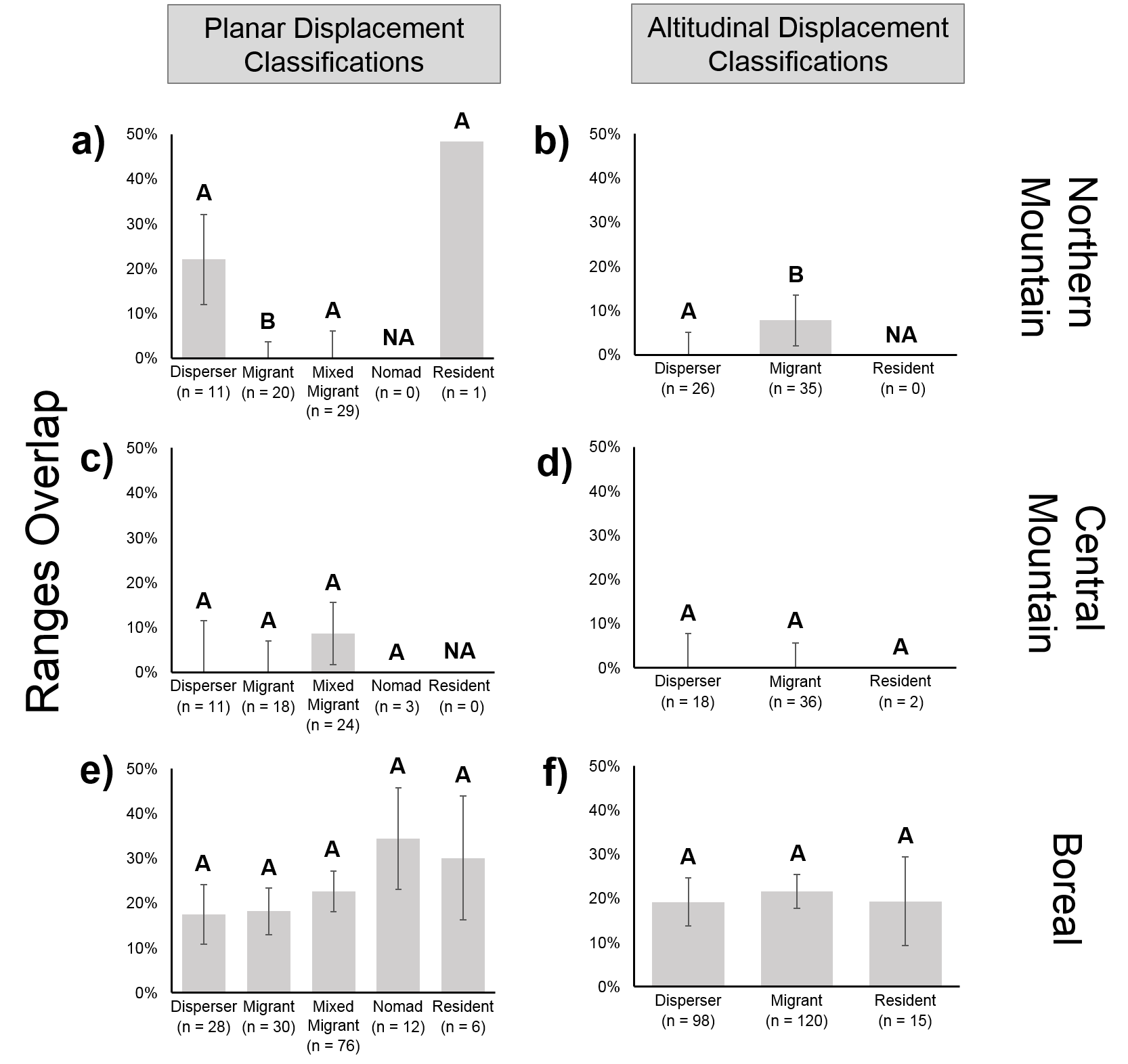


**Fig. S1** Comparisons of seasonal ranges overlap values between each seasonal movement category obtained with displacement analyses. Panels **a** and **b** show comparison of overlap between categories obtained with planar or altitudinal displacement analyses conducted for Northern Mountain caribou. Panels **c-d** and **e-f** show comparisons for Central Mountain and Boreal caribou, respectively. The upper-case letters A/B denote significant differences. Sample sizes for seasonal movement analyzed are reported (n), as well as the 95% confidence intervals.
